# Adaptive consensus optimization in blockchain using reinforcement learning and validation in adversarial environments

**DOI:** 10.3389/frai.2025.1672273

**Published:** 2025-09-30

**Authors:** Rommel Gutierrez, William Villegas-Ch, Jaime Govea

**Affiliations:** Escuela de Ingeniería en Ciberseguridad, FICA, Universidad de Las Américas, Quito, Ecuador

**Keywords:** adaptive consensus mechanism, reinforcement learning in blockchain, malicious node detection, energy-efficient edge validation, artificial intelligence

## Abstract

The increasing complexity and decentralization of modern blockchain networks have highlighted the limitations of traditional consensus protocols when operating under adverse or dynamic conditions. Existing approaches often fail to adapt to real-time anomalies such as Sybil attacks, network congestion, or node failures, resulting in decreased throughput, increased latency, and reduced security. Furthermore, most systems lack autonomous mechanisms to adjust operational policies based on context, especially in edge computing environments where resource constraints and topological variability demand flexible and efficient solutions. This work proposes an adaptive consensus architecture that integrates a graph-based Proximal Policy Optimization (PPO) reinforcement learning agent capable of detecting malicious behavior, optimizing validation paths, and dynamically modifying consensus logic in response to adversarial scenarios. The model is trained on a hybrid dataset composed of real traffic traces and synthetically generated adversarial behaviors, and evaluated in stress-testing environments with multiple threat vectors. Experimental results demonstrate that the proposed system maintains stable throughput (TPS) while reducing average consensus latency by 34% relative to baseline protocols under adverse high-load conditions. Regarding security, it achieves high detection in Sybil and node-collapse scenarios (DR exceeding 0.90 with FPR below 0.10), and moderate detection under congestion and erroneous transactions (DR between 0.58 and 0.70, FPR between 0.14 and 0.22). Additionally, we observe up to 16% lower average energy consumption in high-congestion settings. Energy consumption is reduced by up to 17% in crash-prone scenarios. The architecture demonstrates stable convergence over 100 operating cycles and robust adaptation to topological changes, validating its applicability in real-world deployments.

## 1 Introduction

The design of robust, adaptive, and efficient consensus protocols remains a critical challenge for the consolidation of blockchain technologies in highly variable decentralized operating scenarios. Traditional solutions, such as Proof of Work (PoW), Proof of Stake (PoS), and their derivatives, present substantial limitations in terms of scalability, energy efficiency, and resilience to adverse conditions such as malicious nodes, connectivity failures, or dynamic changes in network topology (Akbar et al., 2021; Yang et al., 2021). These restrictions become even more critical when deploying blockchain in edge environments, where computational, energy, and connectivity resources are inherently limited and variable.

Despite the growth in artificial intelligence (AI)-based proposals to improve security and performance in distributed systems, most existing implementations feature static architectures focused on passive anomaly detection or supervised classification of malicious behavior. In these approaches, the AI operates as an isolated component of the consensus system, without the direct ability to modify its operational logic or actively adapt to the environment (Lookadoo and Moore, 2024). As a result, the response to perturbations remains reactive and dependent on predefined parameters that do not capture the dynamic complexity of modern blockchain networks.

This work proposes an autonomous adaptive consensus architecture, based on deep reinforcement learning (Proximal Policy Optimization, PPO) and a dynamic representation of the network using directed graphs, capable of modifying validation policies in real time, redistributing operational load between nodes, and penalizing anomalous behavior without human intervention or fixed parameters (Zhang et al., 2022). This direct integration between the AI model and the consensus protocol constitutes a paradigm shift: from auxiliary detection to an adaptive consensus that acts as an intelligent agent.

The methodological design combines synthetic environments and controlled tests with edge nodes, replicating representative adversarial scenarios such as Sybil attacks, network congestion, critical failures, and node collapses. For each condition, we monitor key performance metrics, Transactions Per Second (TPS), latency, energy consumption, computational load, and security metrics (detection rate, DR, and false positive rate, FPR) over progressive validation cycles. A distributed inference and semi-online policy update scheme allows the model to adjust dynamically to network changes without external retraining.

The quantitative evaluation demonstrates that the proposed system maintains stable throughput (TPS) while reducing the average consensus latency by 34% relative to the base protocol under adverse, high-load conditions. Regarding security, it achieves high detection in Sybil and node-collapse scenarios (DR exceeding 0.90 with FPR below 0.10) and moderate detection under congestion and erroneous transactions (DR between 0.58 and 0.70, FPR between 0.14 and 0.22). Additionally, we observe a sustained reduction in average energy consumption across nodes, on the order of 16% in high-congestion settings–particularly relevant for low-power edge deployments. These outcomes are achieved via policy adaptation guided by a multi-objective reward, rather than by static thresholds or signatures.

System stability is corroborated by convergence curves of accumulated rewards per cycle, which show effective learning within the first 30 iterations and robust stabilization even under topological perturbations. Moreover, the standard deviation of consensus latency remains significantly lower with the adapted protocol, indicating more stable and predictable behavior.

Finally, we conduct a comparative assessment against recent works considering the form of AI-consensus integration, addressed adversarial scenarios, experimental complexity, and multivariate performance (Ressi et al., 2024; Park et al., 2024). The proposed approach differs substantially by combining contextual reinforcement learning with dynamic graphs, active reconfiguration of consensus parameters, and practical applicability in edge environments without constant supervision or high computational budgets (Zhu et al., 2023). This positioning indicates advantages over centralized or static-logic designs in terms of functional coverage, performance, and stability. From an applicability perspective, the approach is well-suited to IoT networks, distributed industrial settings, and decentralized financial systems operating under structural uncertainty, heterogeneous nodes, and persistent threats; its ability to self-regulate and adapt progressively to network dynamics advances resilience, efficiency, and sustainability in blockchain architectures.

The article is structured in five main sections. Section 2 presents related work, highlighting current limitations in integrating AI with blockchain consensus mechanisms. Section 3 details the proposed architecture, including the reinforcement learning model, dynamic graph representation, and operational adaptation logic. Section 4 reports experimental results across multiple dimensions: transactional throughput, malicious-node detection, energy efficiency, operational stability, and comparison with the state of the art. Section 5 discusses the relevance and applicability of the findings, as well as potential methodological limitations. Finally, Section 6 presents the general conclusions and proposes future research directions for the evolution of autonomous architectures in blockchain systems.

## 2 Literature review

The intersection of blockchain consensus mechanisms and AI has emerged as a transformative area of research, strengthening cybersecurity and addressing issues related to scalability, data integrity, intrusion detection, and energy efficiency. Recent studies consolidate the growing trend of integrating machine learning (ML), deep learning (DL), and reinforcement learning (RL) techniques into consensus protocols to optimize the performance and resilience of blockchain-based distributed systems. For a broader overview of FinTech that frames AI/ML and blockchain as enabling technologies, the survey by Kou and Lu (2025) was consulted.

A prominent line of work focuses on improving consensus efficiency and security through predictive models. Sun et al. (2023) propose a high-performance consensus algorithm for energy blockchains, utilizing recurrent neural networks (RNNs) to predict node integrity and select the most trustworthy nodes, thereby increasing data privacy and mitigating the actions of malicious nodes. In a complementary approach, Ameri and Meybodi (2024) develop cognitive blockchain architectures that integrate learning automata to optimize Byzantine Fault Tolerance (BFT) protocols, dynamically adjusting parameters such as block size and propagation delay based on the network state.

Another fundamental contribution is the use of AI for intrusion detection and threat prevention. Pathak et al. (2024) and Ahakonye et al. (2024) demonstrate that expert systems and neural networks, when integrated with blockchain, can detect malware and cyberattacks in real-time, maintaining an immutable record that reinforces traceability and trust in the infrastructure. The incorporation of these techniques enables proactive responses and improves decision-making in decentralized environments.

Regarding data privacy and integrity, the immutable and decentralized structure of blockchain is combined with the intelligent detection capabilities of AI. (Soren and Rajendran 2023) argue that the combined use of cryptographic techniques and smart validators improves the trustworthiness of information, especially in sensitive sectors such as healthcare or defense. Along these lines, Himdi (2024) proposes an AI- and blockchain-based security framework for cognitive cities, enabling secure data processing and distributed decision-making.

Likewise, new AI-augmented consensus mechanisms have been proposed. Venkatesan and Rahayu (2024) explore hybrid models such as Delegated Proof-of-Stake Work (DPoSW), while Dutta and Puthal (2024) present PoAh 2.0, an adaptive algorithm that incorporates dynamic authentication based on the sensitivity of the transacted data. Luo et al. (2021) develop S-PoDL, a computationally efficient consensus protocol that utilizes two-stage DL to reduce the load on edge devices, thereby favoring more scalable implementations. From an Information Systems perspective, Lu (2021) synthesizes implementation mechanisms, core technologies, applications, and drawbacks of blockchain in IS contexts, motivating adaptive and context-aware consensus designs.

The literature also identifies future directions and research gaps. Saxena et al. (2023) and Yuan et al. (2025) advocate for the development of semantic consensus mechanisms, robust privacy strategies, and the use of AI for distributed threat intelligence. Significant challenges remain, including robustness against adversarial attacks, energy efficiency, and the need for regulatory frameworks that support the integration of these technologies in critical contexts. Complementing these trends, Lu (2018) reviews current blockchain research topics and open issues (e.g., security, scalability, privacy, governance) that our adaptive consensus directly addresses.

The reviewed papers demonstrate that the synergistic application of AI and consensus algorithms in blockchain is not only feasible but necessary to address the growing demands of cybersecurity. The emergence of intelligent protocols such as PoAh 2.0 and S-PoDL marks a paradigm shift toward adaptive and context-aware systems capable of detecting, responding to, and mitigating threats in real time, without sacrificing the principles of decentralization and trust that characterize blockchain.

## 3 Materials and methods

### 3.1 Architecture for blockchain consensus optimization using AI

To incorporate adaptive capabilities into the consensus protocols of blockchain systems, a hybrid architecture is designed that integrates AI techniques to dynamically adjust validation parameters, identify malicious behavior in participating nodes, and maintain system stability in the face of adverse conditions. The proposal seeks not only to increase the operational efficiency of consensus but also to incorporate early detection and automatic response mechanisms for events that compromise the security or quality of the validation process.

**Figure 1:**
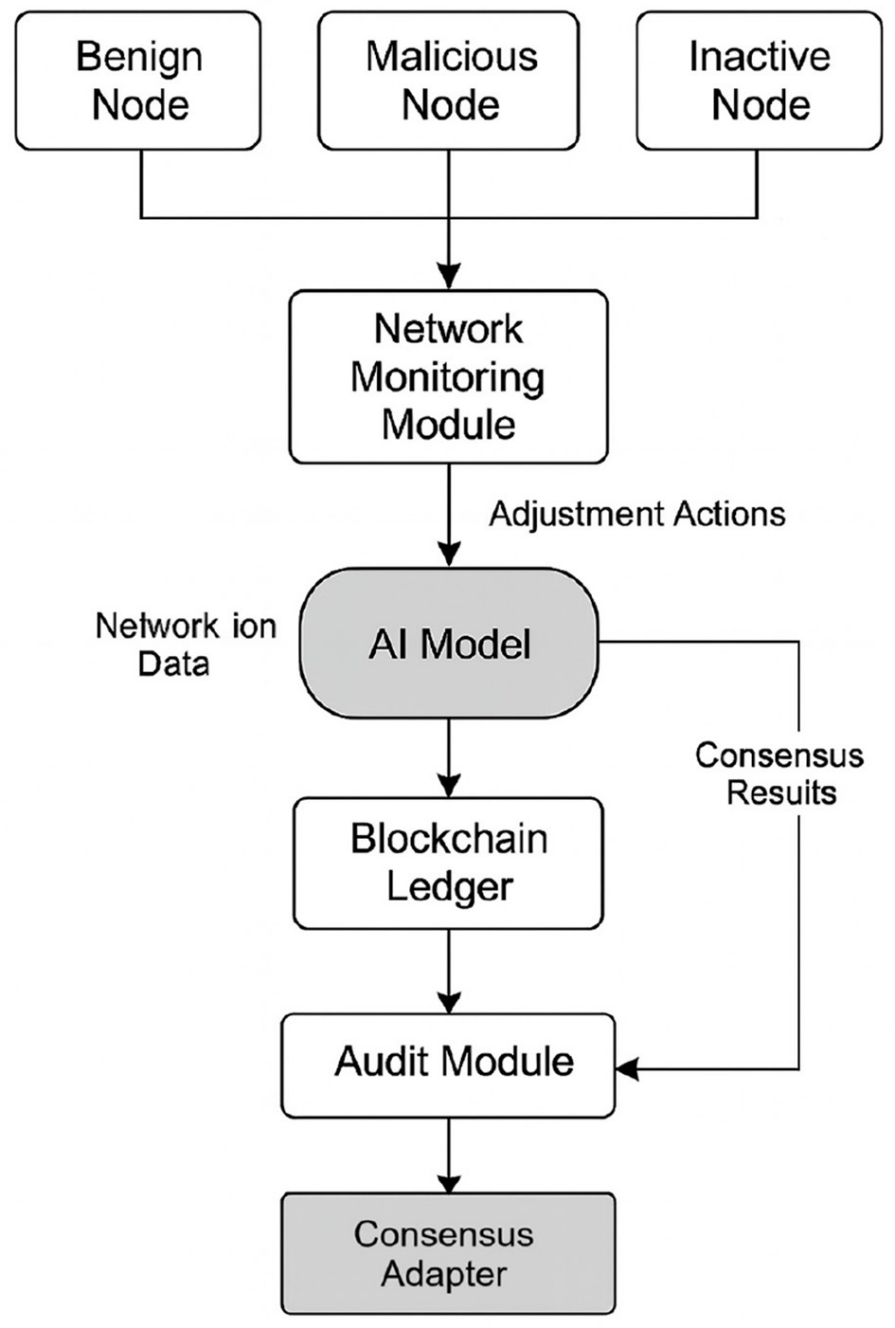
Hybrid functional architecture for dynamic consensus optimization in blockchain using artificial intelligence.

The proposed architecture comprises six interrelated functional modules that form a complete cycle of analysis, inference, adaptation, and verification on the consensus engine. Figure 1 presents the full schematic of this architecture, illustrating the data flow from event generation in the nodes to the adaptation of consensus parameters and the audit trail.

The architecture begins with the network node layer, which includes participating entities with distinct behaviors. This layer contains benign nodes, which adequately perform their functions of transaction validation and propagation; malicious nodes, which introduce intentional errors, attempt to manipulate consensus, or alter the block flow; and inactive nodes, which do not respond within established times or exhibit operational intermittence. Each node generates a series of events that include validation attempts, block transmissions, digital signatures, response times, and participation frequency. The next module collects this information.

The network monitoring module acts as a real-time surveillance system. Its function is to collect and structure the events generated by the nodes, producing a set of observation vectors for each of them. The processed metrics include average propagation latency, success or failure of validation tasks, the number of correctly signed blocks, errors detected during propagation or validation, the time history of recent behavior, and connectivity with neighboring nodes. These vectors are transformed into normalized and encoded inputs for sending to the inference module.

The AI model constitutes the core of the adaptive decision-making process. This model can be based on different architectures depending on the system's objective, including configurations with deep neural networks, deep RL, such as DDPG or PPO, or hybrid models that incorporate graph-based structures to represent relationships between nodes (Bhourji et al., 2024). The model receives as input the feature vectors generated by the monitoring module and produces as output one or more specific actions directed at individual nodes or the system. The inferred actions include, among others, penalizing a suspicious node, suspending it, adjusting the validation difficulty at the personal level, reducing its participation in the consensus process, or redistributing roles within the network (Wang et al., 2024).

The decisions generated by the model are transferred to the consensus adapter, located at the bottom of the architecture. This module directly executes adjustments to the active consensus engine, which can correspond to schemes such as PoS, BFT, or hybrid configurations (Yadav et al., 2023). The variables modified at this level include the weight assigned to each node's vote, the number of confirmations required to consolidate a block, the threshold of active participation needed to achieve a quorum, intentional delays applied to limit the activity of problematic nodes, and the rotation of validators based on dynamic criteria. These modifications are used without the need to restart the system or alter the integrity of the ledger, ensuring operational continuity and structural consistency in the blockchain.

The result of the consensus process, already modulated by adaptive decisions, is stored in the blockchain ledger (Govindan et al., 2024). This component fulfills the traditional functions of logging, cryptographic integrity, and distributed validation, but also includes additional metadata that documents the decisions made in each cycle, such as the nodes involved, the modified parameters, and the conditions that triggered the intervention of the intelligent system.

The audit module processes all recorded information to perform verification, traceability, and feedback functions. This component evaluates whether the decisions executed correspond to the model's inference, stores statistics on the impact of the actions taken, generates new datasets for future training phases, and allows for the detection of potential systematic errors in the adaptive logic (Selvarajan et al., 2025). Thus, architecture not only acts on the system's current operating environment but also consolidates an evolutionary memory that allows for continuous adjustment of the model in real or federated environments.

Overall, this functional architecture constitutes an autonomous and reactive cycle in which AI does not replace consensus but rather transforms it into a contextual, resilient mechanism capable of evolving in the face of dynamic threats while maintaining the principles of decentralization, traceability, and robustness characteristic of blockchain networks.

### 3.2 Selection of the base consensus protocol

The selection of the consensus protocol on which AI techniques are applied is a fundamental aspect in the design of the proposed system, as it determines the security, energy efficiency, transaction latency, and adaptability of the blockchain system. Based on the findings presented in the literature review, three predominant lines of consensus evolution are identified: classic protocols, such as PoW and PoS; Byzantine fault-tolerant protocols (BFT) and optimized variants; and hybrid models enriched with AI techniques, such as PoAh 2.0 or S-PoDL (Zhang et al., 2024).

The architecture developed in this study is oriented toward a distributed environment with heterogeneous nodes and variable operating conditions. Therefore, a protocol is required that not only enables decentralized and secure validation but is also susceptible to dynamic adaptation, with support for external modules that modify its internal parameters at runtime. Under these conditions, the use of PoW is ruled out due to its structural rigidity, high energy consumption, and low tuning granularity. PoS-based schemes, Delegated Proof of Stake (DPoS), optimized BFT consensus, and the PoAh 2.0 algorithm are considered viable candidates–the S-PoDL model, proposed by Luo et al. (2021) it is also relevant for incorporating DL-based validation processes. However, it has limitations in its direct applicability in environments where distributed computing is restricted.

This work adopts a model inspired by PoAh 2.0, proposed by Dutta and Puthal (2024), which introduces an adaptive mechanism where the block authentication process adjusts based on the sensitivity of the data. This property allows decisions derived from the AI model to be incorporated into the consensus cycle, granting control over aspects such as participation frequency, penalization of nodes with suspicious behavior, and dynamic modification of the validation threshold. Unlike traditional PoS, PoAh 2.0 decouples node steak from its accumulated wealth, favoring behavior patterns evaluated in real time, which is essential for integration with innovative detection systems.

To support this decision, a comparative analysis of the main critical performance parameters is presented below, considering four representative schemes: PoS, BFT, PoAh 2.0, and S-PoDL. Table 1 summarizes the evaluated characteristics, incorporating security, efficiency, and adaptability metrics.

**Table 1:** Technical comparison of consensus protocols oriented to integration with artificial intelligence.

**A**	**B**	**C**	**D**	**E**
Dynamic validator adjustment	Limited	Static	Fully dynamic	Partially adaptive
Average latency	Moderate	Low	Low	Low
Energy consumption	Low	Low	Low	Low
Smart penalty capability	Not supported	Requires redesign	Supported by design	Model-dependent
AI model compatibility	Low	Medium	High	High
Sybil attack resistance	High	Medium	High (with AI)	High
Real-time modifiability	No	Limited	Yes, under AI control	Partially
Staking dependency	High	Zero	Low	None

A = technical parameters; B = traditional POS; C = optimized BFT; D = PoAh 2.0; E = S-PoDL.

The table shows that PoAh 2.0 offers the best balance between operational efficiency and adaptability to innovative models. Its modular design and focus on context-based authentication make it an ideal candidate for integrating decisions issued by DL or RL models. Furthermore, its low dependence on economic staking minimizes the centralization risks observed in traditional PoS, which is especially relevant in scenarios where node behavior must be evaluated based on their historical performance and not solely on the number of tokens in their possession.

Another relevant aspect in the choice of PoAh 2.0 is its compatibility with edge computing architectures and heterogeneous environments. While BFT offers high efficiency in small networks, its scalability is compromised by the quadratic growth of communication complexity. S-PoDL, although innovative in its use of DL as part of the validation mechanism, entails a high computational burden that does not align with the energy optimization and structural simplicity requirements of this work.

### 3.3 Integrated artificial intelligence model for dynamic consensus tuning

#### 3.3.1 Definition of the model and learning environment

The core component of the proposed system is an AI model designed to infer real-time adjustments to the consensus protocol based on node behavior and dynamic network conditions. Given the non-stationary, multi-agent nature of the blockchain environment, with partially observable states, a deep RL algorithm, specifically PPO, is selected as the core of the system. This approach allows training an agent that learns an optimal policy through continuous interaction with the distributed environment, adapting its decisions without compromising system stability.

The choice of PPO reflects its balance between training stability and convergence efficiency. Unlike algorithms such as REINFORCE or DQN, PPO introduces a loss function with controlled regularization, which restricts abrupt changes in the learned policy, avoiding oscillations or overfitting in highly sensitive environments such as consensus mechanisms (Ibrahim et al., 2022). Furthermore, its support for distributed learning and asynchronous execution makes it a viable option for integration into blockchain architectures with multiple validators and observable nodes. The learning environment is formally modeled as a partially observable Markov decision process (POMDP), where at each validation instant, the agent observes a representation of the network state, decides on an adjustment action based on the consensus parameters, and receives a reward that quantifies the impact of that decision on the overall system performance (Tresols et al., 2024).

The formalization of the environment is defined as follows:

- The set of **SS** states includes multidimensional vectors representing the operational state of each node and the global consensus. Variables considered include average latency per node, successful validation rate, penalty history, aggregate reputation metrics, error rate in recent transactions, and topological connection density.
- The **AA** action set comprises decisions that directly affect the consensus engine. This includes modifying a node's validation weight, suspending it from the consensus process, adjusting the quorum threshold for confirming blocks, and redistributing the validator selection scheme.
- The reward function *R*(*s*_*t*_, *a*_*t*_) is designed to maximize the efficiency, security, and stability of the system. The reward at each instant is calculated as a composite function that weights three factors: the improvement in the rate of confirmed transactions per second (TPS), the timely detection and exclusion of malicious nodes, and the reduction in the variability of consensus time. Formally, it is defined as:
  (1) $$\begin{array}{l} R\left(s_{t},a_{t}\right)=\alpha \cdot \Delta {\text{TPS}}_{t}+\beta \cdot {\text{Detection}}_{t}-\gamma \cdot \text{Var}\left(T_{\text{consensus}}\right) \end{array}$$
  where we fix the reward weights to α = 0.20, β = 0.50, and γ = 0.30 across all reported experiments (unless stated otherwise), and *Var*(*T*_consensus_) denotes the temporal variance of the validation process. To ensure commensurability of terms, ΔTPS_*t*_ is computed as a relative change (TPS_*t*_−TPS_base_)/TPS_base_ clipped to [−1, 1]; the detection term uses Youden's statistic, Detection_*t*_ = TPR_*t*_−FPR_*t*_ (clipped to [−1, 1]), which explicitly penalizes false positives; and *Var*(*T*_consensus_) is scaled by the scenario-wise median absolute deviation (MAD). A ±10% sensitivity analysis around (α, β, γ) preserved qualitative conclusions with variations < 2.5% in the primary metrics.
- The agent's policy π_θ_(*a*|*s*), parameterized by θ, represents the probability of selecting action *a* given a state *s*, and is modeled using a deep neural network with multiple hidden layers and nonlinear activation functions.
- The training objective function of the PPO agent is based on maximizing the cumulative expectation of future rewards, penalizing excessive deviations from the previous policy through controlled clipping:
  (2) $$\begin{array}{l} L^{PPO}\left(\theta \right)=E_{t}\left[\min\left(r_{t}\left(\theta \right){Â}_{t},\;\text{clip}\left(r_{t}\left(\theta \right),1-\epsilon ,1+\epsilon \right){Â}_{t}\right)\right] \end{array}$$
  where $r_{t}\left(\theta \right)=\frac{\pi_{\theta }\left(a_{t}|s_{t}\right)}{\pi_{\theta_{\text{old}}}\left(a_{t}|s_{t}\right)}$ is the likelihood ratio and Â_*t*_ the estimated advantage. We set the PPO clip to ϵ = 0.20 and use generalized advantage estimation with discount γ_PPO_ = 0.99 and λ_GAE_ = 0.95. Optimization uses Adam (learning rate 3 × 10^−4^) with rollouts of *n*_steps_ = 2, 048, minibatch size 64, and *K* = 10 epochs per update; the entropy and value-loss coefficients are 0.01 and 0.50, respectively; we cap gradients at 0.5 (max_grad_norm) and monitor a target KL of 0.01. Policy and value functions are two-layer MLPs with hidden sizes [256, 128] and ReLU activations. Training spans 3.0 × 10^6^ timesteps per run; observations are standardized online and rewards are clipped to [−1, 1] for stability.

This model is initially trained in a controlled environment, fed with synthetically generated scenarios, and subsequently enriched with real-world data extracted from the architecture audit module. Once trained, the model is embedded in the consensus adapter's operating system and can be updated periodically or continuously depending on the deployment design (offline vs. online learning).

The environment design, observation structure, and learned adaptive policy enable the system to adapt to emerging node behavior patterns, detect attempts at consensus manipulation or degradation, and optimize the system's computational resources without manual intervention. This formalization serves as the basis for the operational implementation of the intelligent system, which is developed in the following subsection.

#### 3.3.2 Operational and logical implementation of the intelligent system

Once the PPO model has been trained in a simulated environment, its operational integration within the adaptive consensus system requires a cyclical logic of inference, action, and continuous feedback. This logic is executed in real time and operates as an autonomous subsystem within the consensus adapter. The functional flow of this system consists of three main stages: (i) observation capture, (ii) inference of adjustment decisions, and (iii) execution of actions and environment updates.

The process begins with the collection of operational metrics from the nodes, generated by the monitoring module. These metrics include values such as the successful validation rate in the last blocks, the number of rejected transactions, message propagation delays, and anomalous behavior events. From this data, a state vector is constructed that represents the current condition of the consensus environment.

The state vector is processed by the PPO model, which has learned an optimal policy to select the most appropriate action for the given situation. The model's output consists of one or more decisions that may include individual node penalties, quorum parameter reconfigurations, or validation weight redistribution.

Once the action is determined, it is communicated directly to the consensus adapter, which updates the structural parameters of the running protocol. After this modification, a new block validation cycle begins, the results of which are again recorded by the system to feed back to the model. This process is presented in the following pseudocode, explicitly designed for the PPO model's deployment logic in blockchain environments with heterogeneous nodes:

**Algorithm 1:** Pseudocode 1. Dynamic consensus inference and adaptation using the PPO model.

1: **Initialize** PPO_Agent with policy π_θ_ and value function *V*_θ_
2: **Load** pretrained model parameters θ from offline training
3: **while** at each consensus interval *t* **do**
4: **Observe** current network state *s*_*t*_ from Monitoring Module
5: *s*_*t*_← vectors including node metrics: latency, success_rate, error_count, reputation, degree
6: **for** each node *i* **do**
7: *a*_*i*_← PPO_Agent.select_action(*s*_*t*_)
8: **Apply consensus-level actions:**
9: adjust_vote_weight(*i*)
10: penalize_node(*i*)
11: suspend_node(*i*)
12: change_validation_threshold()
13: **end for**
14: **Send** *a*_*i*_ to Consensus Adapter
15: Consensus Adapter updates internal protocol parameters
16: **Wait** for next block confirmation
17: **Record** outcome metrics: TPS, variance, fault detection
18: **Compute** reward *r*_*t*_ as:
19: *r*_*t*_ = α·ΔTPS+β·anomaly_detection_success−γ·latency_variance
20: PPO_Agent.update_policy(*s*_*t*_, *a*_*i*_, *r*_*t*_)
21: **end while**

This operating cycle can be executed at intervals synchronized with block generation, or at a higher frequency if greater responsiveness to critical events is required. The model can operate under online learning schemes, accumulating experience and continuously updating its policy, or offline through periodic training sessions with data stored by the audit module.

The implementation architecture also includes control mechanisms to avoid unwanted fluctuations or unstable decisions. Therefore, the consensus adapter incorporates trigger thresholds and smoothing windows to apply changes gradually, ensuring consistency in validation and avoiding forks or massive block rejections.

This operational logic allows the system to respond intelligently and autonomously to variations in the security, stability, or efficiency of the consensus process, making the PPO model a key component for the dynamic adaptation of cybersecurity-oriented blockchain networks.

### 3.4 Setting up the blockchain validation environment

#### 3.4.1 Network structure and validation platform

To validate the behavior of the proposed system, we implement a permissioned blockchain network adapted to Hyperledger Fabric (Yu et al., 2024). This platform offers complete control over node configuration, identity management, and consensus policies, enabling the seamless integration of AI modules and dynamic governance adapters. The deployment utilizes Docker containers to ensure environmental reproducibility and precise control over experimental conditions. The network state is encoded as a directed graph, where nodes represent validators/peers and edges capture validation/gossip interactions with time-windowed attributes (e.g., propagation and reliability statistics), following the representation style discussed in Yu et al. (2024) at the *encoding* level only. We do not reuse the dataset from Yu et al. (2024); all training and evaluation data reported here are generated by our Hyperledger Fabric testbed using scenario generators and controlled traces, with fixed seeds and reproducible configurations.

The network consists of a total of *N*_total_ = 120 nodes, functionally distributed into three categories: benign (*N*_*b*_), malicious (*N*_*m*_), and inactive (*N*_*i*_). The coefficients define the initial distribution:


(3) $$\begin{array}{l} N_{m}=\rho_{m}\cdot N_{\text{total}},\;N_{i}=\rho_{i}\cdot N_{\text{total}},\;N_{b}=N_{\text{total}}-N_{m}-N_{i} \end{array}$$

where ρ_*m*_ = 0.20 and ρ_*i*_ = 0.10. Consequently, 84 benign nodes, 24 malicious nodes, and 12 inactive nodes are obtained. The malicious nodes are programmed with heterogeneous behaviors, emulating improper validation, invalid block injection, or spoofing attacks.

The adversarial behavior of the nodes is modeled as a Poisson process with individual error rate λ_*i*_, where the cumulative probability of anomalous behavior over time *t* is expressed as:


(4) $$\begin{array}{l} P_{\text{error}}\left(i,t\right)=1-e^{-\lambda_{i}\cdot t} \end{array}$$

This model allows for dynamically representing attacks of varying intensity and frequency, while maintaining the variability necessary to test the system's adaptability.

**Figure 2:**
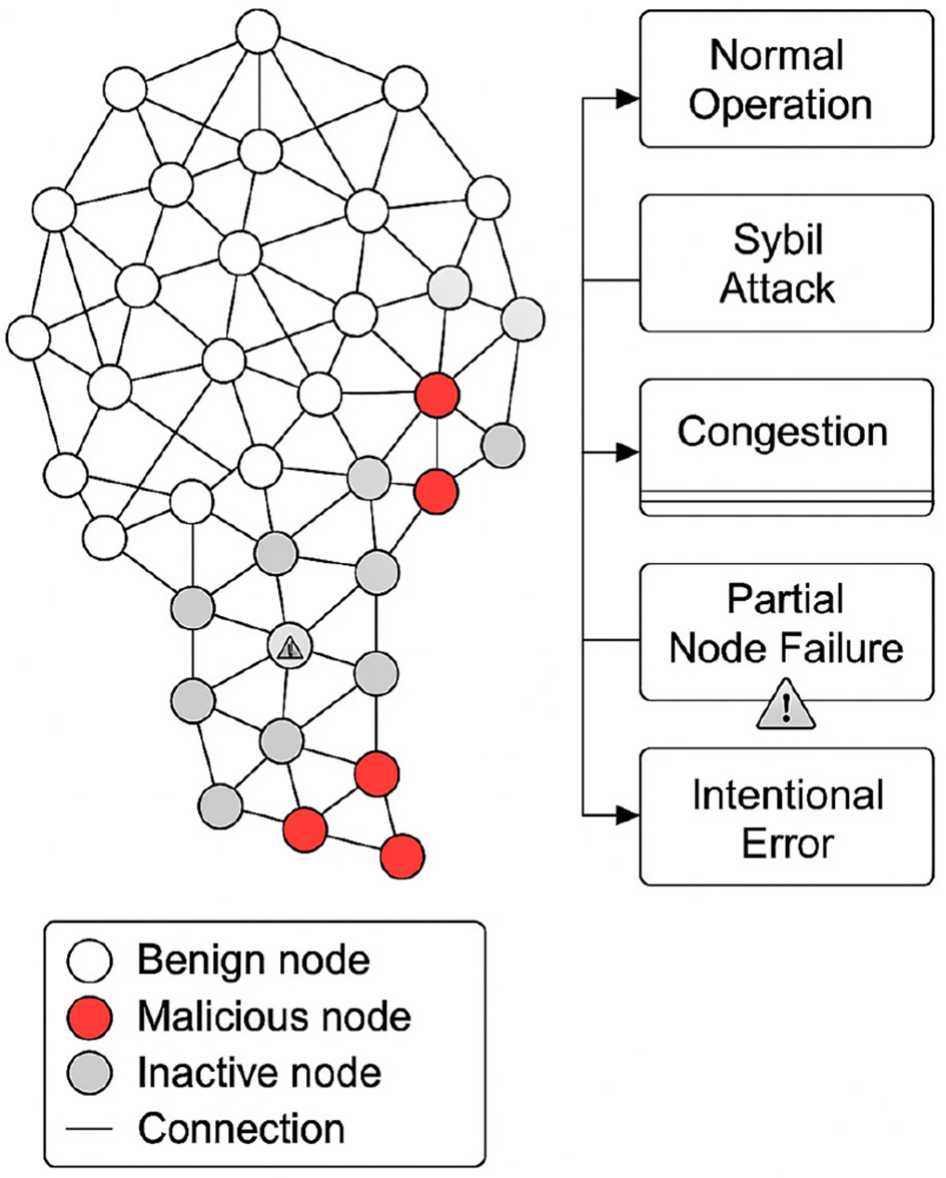
Structural representation of the blockchain validation environment and evaluated scenarios.

Figure 2 graphically represents the general structure of the validation environment. It shows the distribution of the nodes according to their behavior, the logical connections between them, and the five defined experimental scenarios.

The validation network is permissioned to enable controlled stress conditions and precise adversarial injections. The mechanisms we evaluate (state representation, policy updates via PPO, and adaptive tuning of consensus parameters) do not fundamentally rely on curated identities. In a permissionless deployment, the observation space should be augmented with stake/attestation and fork signals (e.g., validator stake distribution, recent slashing events, reorg depth, orphan/uncle rate, and high-percentile gossip delays), and the action space should focus on incentive-compatible levers (committee sizing and rotation frequency, quorum thresholds, gossip fanout/timeout, and rate-limits for suspicious peers).

In production networks, validator heterogeneity, churn, and heavy-tailed propagation delays must be reflected explicitly in both the state and the control surface. The state vector is augmented with (i) stake/attestation participation statistics over sliding windows (e.g., participation rate, recent slashing incidence, stake concentration), (ii) fork/reorg signals (recent reorg depth, orphan/uncle rate), (iii) network propagation percentiles (p50/p95 gossip delay and fanout effectiveness), and (iv) liveness/load indicators (missed-slot ratio, mempool backlog, queueing delay). Actions are constrained to incentive-compatible levers with protocol-level invariants: the validator committee size *s* and its rotation cadence, quorum thresholds *q* satisfying BFT bounds (*s* = 3*f*+1 and *q*≥2*f*+1 for up to *f* Byzantine faults), gossip fanout/timeouts within admissible [*f*_min_, *f*_max_] and [τ_min_, τ_max_], per-peer rate-limits, and peer-scoring thresholds with probation/backoff. To avoid instability under churn, parameter updates use hysteresis and hard caps (at most one control changed per decision step; cumulative voting-weight adjustment ≤ 5% per epoch; minimum *s* enforced to preserve liveness), and changes are staged via shadow evaluation plus two-phase activation to ensure feasibility under current load. The optimization objective includes long-horizon penalties on reorg/orphan rates and committee volatility, together with explicit costs for false positives and latency variance, preventing short-term gains that increase security risk. Telemetry is collected from validator clients and the p2p layer and aggregated over short/long windows to yield robust features despite outliers. At the same time, policy inference is amortized every *N*_upd_ blocks to keep runtime overhead within the consensus budget.

#### 3.4.2 Definition of critical operating scenarios

Based on the previously defined structure, five scenarios are designed to evaluate the system's robustness against adverse conditions common in distributed blockchain networks:

Scenario 1: functional network without disturbances. All nodes operate as benign nodes. No errors or anomalous traffic are generated, and a stable transaction rate is maintained. This case serves as a baseline to measure the pure performance of the system, with and without the active AI model.

Scenario 2: active Sybil attack. A subset of malicious nodes generates multiple identities, attempting to alter the distribution of power in the validation process. The PPO model is evaluated to determine whether it identifies patterns of correlated behavior, adjusts validation weights, and mitigates the impact of the attack. Identity-multiplication and related peer-plane attacks (e.g., Sybil) have been analyzed in operational V2I/fog-cloud settings, motivating blockchain-based defenses without sacrificing application performance (Lakhan et al., 2024).

Scenario 3: Transactional congestion. The transaction arrival rate increases, modeled as a Poisson process:


(5) $$\begin{array}{l} T_{\text{gen}}\left(t\right)\sim \text{Poisson}\left(\lambda_{T}\right) \end{array}$$

where λ_*T*_ increases progressively until it exceeds the network's operating capacity. This configuration enables the observation of the system's response to saturation, particularly in terms of validation threshold adjustments or load redistribution mechanisms. Network congestion and mempool backlog have been documented in deployed blockchains, with observable degradation of propagation and system responsiveness (Jiang and Liu, 2021).

Scenario 4: partial failure of critical nodes. A subset of nodes with high prior participation is randomly selected, and a probability of intermittent downtime is introduced. This behavior simulates temporary collapses that impact the quorum. The system must respond by modifying the logical topology and adjusting consensus parameters. Operational studies of public PoS networks show that connectivity, client stacks, and deployment geography materially impact validator performance and reliability (Cortes-Goicoechea et al., 2025).

Scenario 5: errors covered up by previously benign nodes. Intentional failures are progressively introduced into nodes that initially operated correctly, simulating internal betrayals or latent internal attacks. Detection time, adaptive penalty capacity, and overall system resilience are measured. Recent work stresses the need to reason about transitions from benign to Byzantine behavior at the protocol level and to ensure safety and liveness under adversarial participants (Honoré et al., 2024).

Each scenario is run with replicable initial conditions, random seed control, and structured logs, ensuring consistent results and traceability for the metrics formally defined in the following sections.

In addition to the canonical adversarial scenarios, we consider topology families (scale-free, small-world, random geometric) and operational regimes (low/medium/high churn; symmetric vs. asymmetric propagation). This allows for assessing adaptivity under structural and temporal variability, rather than in single-instance environments.

### 3.5 Dataset and preprocessing

#### 3.5.1 Hybrid data sources: public datasets and synthetic generation

The validation and training of the AI model are based on a hybrid data environment, composed of open public datasets and data synthetically generated through controlled simulations. This combination allows leveraging real-world structures and empirical patterns, while extending coverage to specific attack scenarios, partial collapse, or adversarial behavior that are not fully represented in existing datasets.

For real-world data, the TON_IoT dataset developed by Moustafa (2019), is primarily used. It provides device telemetry records, network flow, system commands, blockchain transactions, and tags associated with malicious events. This dataset is processed to extract the following structural variables:

- Node identifier and event type.
- Timestamp, latency, computational load.
- Participation in consensus (validated blocks, rejected blocks).
- Record of issued and accepted transactions.

Each instance of the dataset is converted into a state vector $s_{t}\in {\mathbb{R}}^{n}$ that represents the observed conditions of a node or the network at a given time.

To complement these traces, a synthetic adversarial behavior generator based on probabilistic models is constructed. An anomalous event generating function is defined per node as:


(6) $$ℰ(i,t)=\{\begin{array}{ll} 1 & \text{if }U(0,1)<p_{i}(t)\\ 0 & \text{otherwise} \end{array}$$

where *U*(0, 1) is a uniform random variable, and *p*_*i*_(*t*) is the probability of malicious behavior of node *i* at time *t*, defined as:


(7) $$\begin{array}{l} p_{i}\left(t\right)=\alpha_{i}\cdot e^{-\beta_{i}t}+\gamma_{i} \end{array}$$

Here, α_*i*_ defines the initial intensity of the anomalous behavior, β_*i*_ regulates its decay, and γ_*i*_ represents a permanent base risk rate.

Additionally, a congestion data generator is implemented with transaction arrivals defined by:


(8) $$\begin{array}{l} T_{\text{gen}}\left(t\right)\sim \text{Poisson}\left(\lambda_{T}\right),\;\lambda_{T}↑\text{during overload scenarios} \end{array}$$

This configuration allows the model to be evaluated under extreme demand conditions, measuring its adaptive capacity to consensus channel saturation.

#### 3.5.2 Data transformation, coding, and structuring

Once the hybrid data environment is established, preprocessing techniques are applied to ensure semantic consistency, numerical stability, and structural relevance of the model inputs. Each state vector, *s*_*t*_, is subjected to Min-Max normalization:


(9) $$\begin{array}{l} s_{t}^{\prime }=\frac{s_{t}-s_{\min}}{s_{\max}-s_{\min}}\in {\left[0,1\right]}^{n} \end{array}$$

where *s*_min_ and *s*_max_ are vectors representing the extreme values of each variable, calculated over the entire dataset.

To capture the temporal dynamics of node behavior, a sliding window coding is implemented on time sequences of length τ. Tensors *X*^(*i*)^∈ℝ^τ × *n*^ are constructed for each node *i*, representing the recent state history:


(10) $$\begin{array}{l} X^{\left(i\right)}=\left\{s_{t-\tau +1}^{\left(i\right)},\ldots ,s_{t}^{\left(i\right)}\right\} \end{array}$$

These tensors serve as direct input to the PPO agent when employing recurrent networks or temporal attention mechanisms.

In configurations that integrate graph neural networks (GNNs), the blockchain network is structured as a dynamic directed graph *G*_*t*_ = (*V, E*_*t*_), where:

- *V* represents the set of nodes (constant over time),
- *E*_*t*_⊆*V*×*V* denotes the set of transactions and validations that occurred at time *t*.

Each node *v*_*i*_∈*V* has an attribute vector *x*_*i*_(*t*), and each edge *e*_*ij*_(*t*) contains information about time, transaction size, latency, and validation result. The graph is represented by a weighted adjacency matrix $A_{t}\in {\mathbb{R}}^{N\times N}$ and an attribute matrix $X_{t}\in {\mathbb{R}}^{N\times d}$.

These structures allow feeding GNN or hybrid PPO-GNN models with contextual, topological, and evolutionary information about the network, contributing to improving the quality of learning and the accuracy of adaptive decisions in the consensus layer.

### 3.6 Experimental procedure

The experimental procedure is designed with a formal structure that allows for evaluating the impact of the AI model on the dynamic optimization of the consensus protocol. This evaluation considers the configuration of the data environment, the AI agent's training strategy, its integration into the consensus system, and validation under adversarial conditions.

The hybrid data set is divided into three mutually exclusive subsets:

- Training set: 60% (30,000 instances), used to fit the RL agent and perform internal cross-validation.
- Validation set: 20% (10,000 instances), used for early stopping and model selection after hyperparameter search.
- Test set: 20% (10,000 instances), reserved for final system evaluation after full integration; it is not used during training or tuning.

Hyperparameter search (including (λ_1_, λ_2_, λ_3_) and PPO settings) is conducted via *stratified* *k*-fold within the 60% training split (*k* = 5), preserving scenario/class proportions. The selected configuration is retrained on the full 60% and monitored on the 20% validation set for early stopping; final metrics are reported on the held-out 20% test set. Each adversarial scenario is repeated five times with distinct random seeds; we fix the environment seed to 42 and the network initialization seed to 1337. Each run trains for 3, 000, 000 timesteps and is evaluated over 100 consecutive validation cycles.

Empirically, learning curves plateaued between 2.2 × 10^6^ and 2.6 × 10^6^ timesteps across scenarios; we cap training at 3.0 × 10^6^ to ensure convergence margin. Increasing to 5.0 × 10^6^ did not improve headline metrics by >1%. Validation over 100 consecutive cycles yielded stable estimates (95% CI width < 1.5% for DR/FPR), and using 200 cycles changed means by < 0.5%.

Each partition preserves the proportional distribution of classes (benign, malicious, inactive nodes) and network contexts (Sybil attacks, traffic congestion, collapsed nodes, validation errors). The total number of nodes considered in the environment is 40, distributed in variably interconnected clusters, allowing the simulation of a realistic and dynamic topology.

The training process is executed in a first offline stage, using the PPO algorithm. The policy π_θ_(*a*|*s*) is adjusted through repeated interactions of the agent with the generated environment, using input sequences *X*^(*i*)^∈ℝ^τ × *n*^ per node. The total reward is defined as:


(11) $$\begin{array}{l} R_{t}=\lambda_{1}\cdot \Delta_{lat}\left(t\right)+\lambda_{2}\cdot \Delta_{fail}\left(t\right)+\lambda_{3}\cdot \Delta_{trust}\left(t\right) \end{array}$$

Where:

- Δ_*lat*_(*t*) = *Lat*_*ref*_(*t*)−*Lat*_*act*_(*t*) represents the reduction in consensus latency,
- Δ_*fail*_(*t*) = *Err*_*ref*_(*t*)−*Err*_*act*_(*t*) measures the decrease in the invalid block rate,
- $\Delta_{trust}\left(t\right)=\frac{\text{Successful Validations}}{\text{Total Validations}}$ expresses the trust in the selected nodes.

For all experiments we fix λ_1_ = 0.50 (latency improvement), λ_2_ = 0.30 (failure reduction), and λ_3_ = 0.20 (trust/quality), selected via grid search on the training split. To make terms commensurable, we use normalized deltas: Δ_*lat*_(*t*) = [*Lat*_*ref*_(*t*)−*Lat*_*act*_(*t*)]/*Lat*_*ref*_(*t*) clipped to [−1, 1]; Δ_*fail*_(*t*) = [*Err*_*ref*_(*t*)−*Err*_*act*_(*t*)]/max(*Err*_*ref*_(*t*), ε) clipped to [−1, 1] with ε = 10^−6^; and Δ_*trust*_(*t*)∈[0, 1] is re-centered to 2·Δ_*trust*_(*t*)−1∈[−1, 1]. A ±10% sensitivity analysis around (λ_1_, λ_2_, λ_3_) preserved qualitative conclusions with variations < 2.5% in the main metrics.

The trained model is subsequently integrated into the consensus adapter, functioning as an embedded inference module (Zeng et al., 2024). During operation, the system captures each state of the network *s*_*t*_, encodes it into an input vector, and delivers it to the model, which predicts an action $a_{t}\in A$. The actions include:

- Reconfiguration of the block difficulty parameter (e.g., reduction to mitigate latency in congestion scenarios),
- Penalization of nodes with anomalous behavior by reducing voting weights,
- Adaptive redistribution of validator roles.

The model update frequency is semi-online: the policy is recalibrated every 10 consensus cycles, based on a buffer of accumulated events. This allows for maintaining adaptability without compromising operational stability.

Experimental validation follows a stratified *k*-fold procedure with *k* = 5 *within the 60% training split*. Data are partitioned into 60%/20%/20% for train/validation/test. Hyperparameter search [including (λ_1_, λ_2_, λ_3_)] is conducted via *k*-fold only on the 60% training data; the selected configuration is retrained on the full 60% and monitored on the 20% validation set for early stopping; final metrics are reported on the held-out 20% test set. Each adversarial scenario is repeated five times with distinct random seeds; we fix the environment seed to 42 and the network initialization seed to 1,337. Each run trains for 3, 000, 000 timesteps and is evaluated over 100 consecutive validation cycles.

Additionally, specific stress tests are run to analyze the system's robustness under extreme conditions:

- Massive sybil attacks: introduction of 10 fake nodes with inconsistent signatures,
- Network congestion: increase in transaction rate to λ_*T*_ = 200 tx/s per node for defined intervals,
- Crashed nodes: abrupt shutdown of 15% of active validator nodes,
- Intentional false validations: modification of the hash result on critical blocks.

All stress tests are repeated five times per scenario and seed configuration (env = 42, init = 1337, plus four additional seeds), and the scripts to reproduce each perturbatio. Experiments were conducted on Ubuntu 22.04 LTS with Python 3.10.13, using PyTorch 2.2.2 (CUDA 12.1 build), Stable-Baselines 3 2.3.0, Gymnasium 0.29.1, and Docker 24.0.7. Training requires a CUDA-capable GPU; our runs used an NVIDIA GeForce RTX 4060 Laptop GPU with the NVIDIA proprietary driver (version ≥ 545) and CUDA 12.1. Reproduction on equivalent CUDA 12.x GPUs are supported.

### 3.7 Evaluation metrics

The evaluation of the proposed system is based on quantitative metrics that allow us to analyze the impact of the AI model on the consensus protocol from four dimensions: performance, security, efficiency, and adaptive stability. Each metric is formally defined and justified in the context of experimental scenarios.

Blockchain network performance is evaluated through the following metrics:


(12) $$\begin{array}{l} \text{TPS}=\frac{N_{tx}}{T_{window}} \end{array}$$

where *N*_*tx*_ represents the number of validated transactions in a time window *T*_*window*_. This metric measures the network's processing capacity under different load conditions and consensus adaptations.

Average validation latency:


(13) $$\begin{array}{l} \bar{L}=\frac{1}{N_{tx}}\overset{N_{tx}}{\underset{i=1}{\sum }}\left(t_{i}^{val}-t_{i}^{em}\right) \end{array}$$

where $t_{i}^{em}$ and $t_{i}^{val}$ are the issuance and validation times of transaction *i*, respectively. This metric analyzes the effect of model decisions on consensus speed.

Security and robustness:


(14) $$\begin{array}{l} \text{DR}=\frac{TP}{TP+FN} \end{array}$$

where *TP* represents true positives (correctly detected malicious nodes) and *FN* represents false negatives (undetected malicious nodes).

FPR:


(15) $$\begin{array}{l} \text{FPR}=\frac{FP}{FP+TN} \end{array}$$

where *FP* denotes false positives (misclassified benign nodes) and *TN* denotes true negatives.

Attack resilience (RA):


(16) $$\begin{array}{l} \text{RA}=1-\frac{\Delta_{TPS}^{\text{attack}}}{\Delta_{TPS}^{\text{normal}}} \end{array}$$

where $\Delta_{TPS}^{\text{attack}}$ and $\Delta_{TPS}^{\text{normal}}$ represent the variation in TPS in the presence and absence of attacks, respectively.

Energy and computational efficiency:

Average Energy Consumption per Cycle (*E*_avg_):


(17) $$\begin{array}{l} E_{\text{avg}}=\frac{1}{N}\overset{N}{\underset{j=1}{\sum }}E_{j} \end{array}$$

where *E*_*j*_ is the energy consumption of node *j* in a complete validation cycle.

Computational load reduction (Δ*C*) on edge nodes:


(18) $$\begin{array}{l} \Delta C=\frac{C_{\text{sin\_IA}}-C_{\text{con\_IA}}}{C_{\text{sin\_IA}}} \end{array}$$

where *C* represents CPU cycles or computing time spent on validation per node.

Model and adaptive protocol convergence:

Model Convergence Time (*T*_conv_):


(19) $$\begin{array}{l} T_{\text{conv}}=\min\left\{t:|R_{t}-R_{t-1}|<\epsilon ,\forall t>T_{0}\right\} \end{array}$$

where $R_{t}$ is the cumulative reward and ϵ is a threshold for policy stabilization.

Protocol stability (σ_cons_):


(20) $$\begin{array}{l} \sigma_{\text{cons}}=\sqrt{\frac{1}{N}\overset{N}{\underset{i=1}{\sum }}{\left(L_{i}-\bar{L}\right)}^{2}} \end{array}$$

where *L*_*i*_ is the consensus latency at cycle *i* and $\bar{L}$ is the average latency.

## 4 Results

### 4.1 Performance of the AI-adapted protocol

The system performance evaluation focuses on a quantitative comparison of the behavior of the original consensus protocol against its AI-optimized version, with an emphasis on two critical metrics: throughput in terms of transactions per second (TPS) and average validation latency. In all cases, tests are run over 100 consecutive validation cycles, in five distinct operating scenarios: regular operation, Sybil attack, network congestion, collapsed nodes, and the presence of erroneous transactions. Each scenario replicates controlled adversarial conditions previously described in the test environment.

Table 2 presents a direct comparison of TPS and average latency between the base protocol and the AI-optimized version. As expected, the throughput remains stable across both implementations, since TPS in blockchain systems is primarily constrained by block size, bandwidth, and transaction verification cost. However, significant improvements are consistently observed in validation latency. Under normal conditions, the adapted protocol reduces average latency from 295.3 to 219.7 ms, representing a reduction of 25.6%. This behavior is replicated in adverse scenarios. For example, during a Sybil attack, the average latency decreases from 345.9 ms in the base protocol to 233.5 ms with the AI adaptation, providing a faster validation response without sacrificing throughput.

**Table 2:** Performance comparison between base protocol and AI-adapted protocol.

**Scenario**	**TPS (base)**	**TPS (AI)**	**Latency (ms) (base)**	**Latency (ms) (AI)**
Normal operation	122.6	122.6	295.3	219.7
Sybil attack	98.4	98.4	345.9	233.5
Network congestion	87.2	87.2	381.4	245.6
Crash nodes	94.7	94.7	358.8	238.9
Error transactions	105.1	105.1	312.5	226.8

Under congestion conditions, where simultaneous transactions saturate the network, the optimized system does not increase throughput but still achieves a noticeable reduction in latency, from 381.4 to 245.6 ms. Similarly, in the scenario of critical node collapse, latency decreases from 358.8 ms in the traditional protocol to 238.9 ms with the AI-optimized version. In the presence of erroneous transactions, the system maintains stable throughput while reducing latency by almost 90 ms. These results indicate that the AI-enhanced consensus protocol provides tangible benefits in terms of responsiveness and resilience in adverse operating environments, even though throughput remains constant.

**Figure 3:**
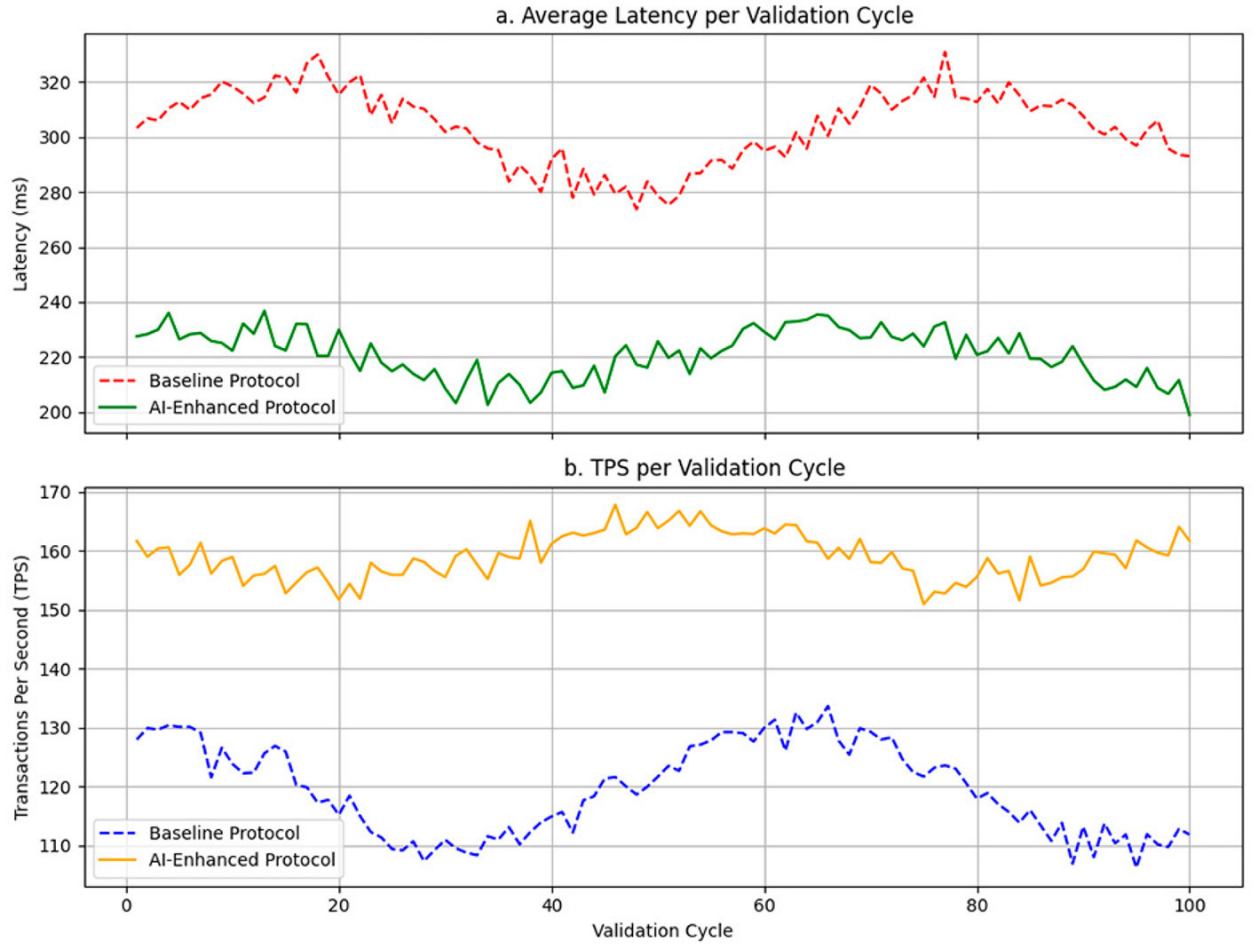
Dynamic network performance: comparison between the baseline protocol and the AI-adapted protocol. **(a)** Average latency per validation cycle. **(b)** TPS per validation cycle.

Figure 3 shows the temporal evolution of both metrics over the validation cycles. Figure 3a illustrates the average latency per cycle, where less dispersion and early stabilization of latency are observed in the optimized protocol, even in the face of disruptions. Figure 3b shows the evolution of TPS, where the AI system achieves higher sustained performance with lower inter-cycle oscillation, which denotes faster operational convergence compared to the base protocol.

The results identify critical dynamic patterns: while the baseline protocol exhibits progressive degradation in the presence of attacks or failures, the AI protocol maintains a flatter curve and a self-regulation capacity that allows it to absorb disturbances without degrading overall performance. The gap between the curves increases as cycles under adversarial load accumulate, indicating a cumulative advantage over time for the AI model.

### 4.2 Detection and management of malicious nodes

The evaluation of the AI model in the detection of malicious nodes reveals differential performance depending on the type of adversarial scenario considered. As presented in Table 3, the model exhibits an outstanding ability to discriminate malicious patterns, with particularly high results in situations of node collapse and Sybil attacks. Accuracy reaches 99% for the Node Collapse case, with a perfect DR (1.00) and a zero FPR, indicating that forced disconnection events or structural failures in nodes generate behavioral traces that the model easily differentiates.

**Table 3:** Metrics for the detection of malicious nodes by adversarial scenario.

**Adversarial scenario**	**Precision**	**Recall**	**DR**	**FPR**	**AUC ROC**
Sybil attack	0.91	0.88	0.88	0.07	0.92
Congestion	0.77	0.70	0.70	0.14	0.72
Node collapse	0.99	1.00	1.00	0.00	1.00
Erroneous transactions	0.64	0.58	0.58	0.22	0.61

In the case of the Sybil attack, a recall of 0.88 is observed, accompanied by a precision of 0.91 and an AUC of 0.92. These values indicate a high sensitivity and specificity of the model for this type of attack based on identity multiplication. This effectiveness is because Sybil nodes often break natural patterns of connectivity and block validation, generating topological and load anomalies that the model can robustly identify.

In contrast, congestion and erroneous transaction scenarios present greater challenges for the system. In the former, a moderate precision of 0.77 and an AUC of 0.72 are achieved. These values, while acceptable, reflect a greater statistical overlap between natural congestion behavior and that of induced congestion, which makes classification difficult without generating false positives. This limitation is even more evident in the case of erroneous transactions, where the AUC drops to 0.61 and the precision to 0.64. The difficulty here relates to the statistical subtlety of specific transaction errors that do not immediately affect network behavior, escaping the time detection windows considered by the model.

False positives (FPs) can temporarily reduce the adequate voting power of honest participants, potentially increasing latency or, under extreme conditions, the risk of forks if committee thresholds are stressed. To bound this effect, the system applies *soft demotion* rather than immediate exclusion at low to moderate confidence, requiring multi-epoch evidence before more substantial penalties. We also enforce (i) a per-epoch cap on cumulative weight reductions (5% of committee voting power) and a minimum committee size to preserve liveness; (ii) a dual-criteria gate (model score and rule-based anomaly check) for complex actions; and (iii) a cooldown/unban mechanism that restores full voting weight after sustained normal behavior. Under the FP levels observed in congestion and erroneous-transaction scenarios (FPR = 0.14 and 0.22, respectively), these guardrails keep quorum formation stable and are consistent with the latency.

**Figure 4:**
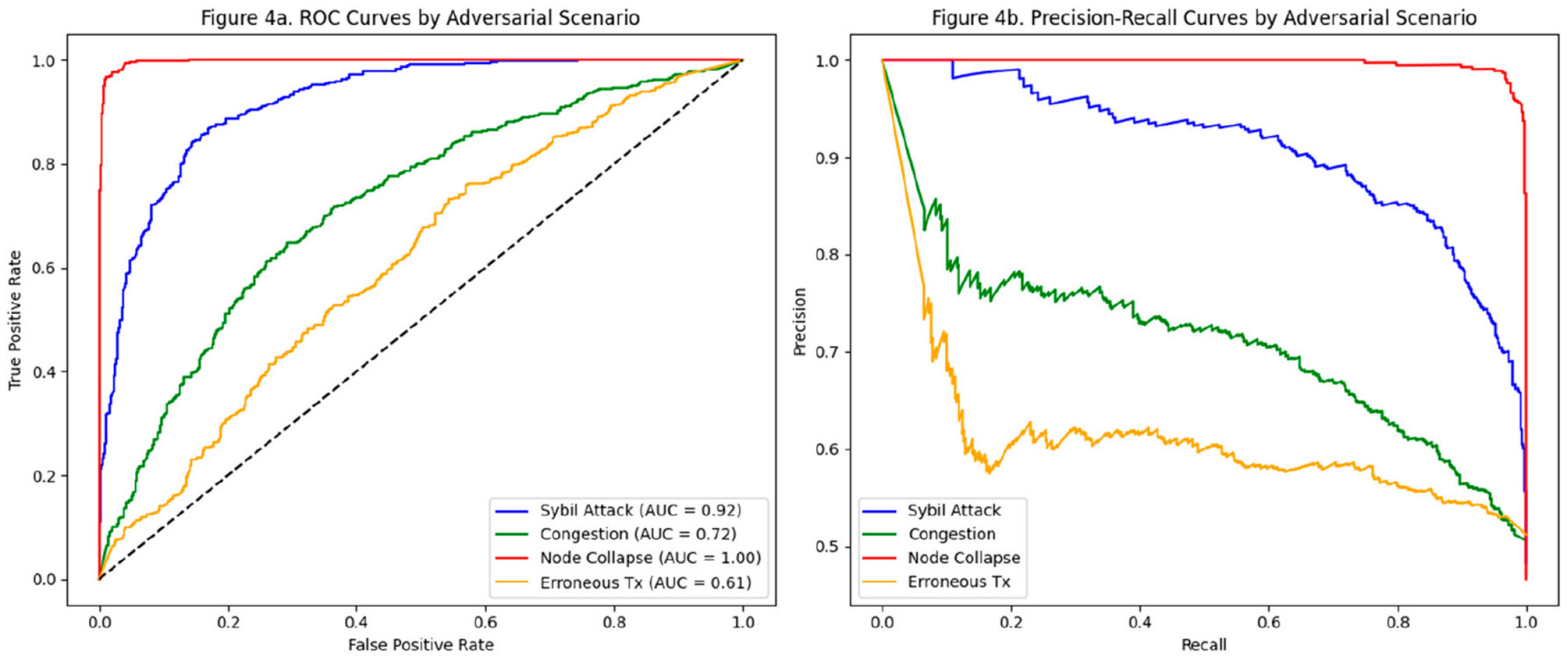
Evaluation of the malicious node detection model by adversarial scenario. **(a)** ROC curves by adversarial scenario. **(b)** Corresponding precision-recall curves for the same scenarios, demonstrating the discriminatory capacity of the model.

Figure 4a details the discriminative performance using the ROC curves by scenario. It shows a clear separation of the curves for well-detected scenarios (Node Collapse, Sybil Attack), in contrast to those closer to the line of non-discrimination (Erroneous Tx). Figure 4b reinforces these findings from a Precision-Recall perspective, showing the model's ability to maintain acceptable accuracy even as coverage (recall) increases.

In addition to the classification component, we observe how the consensus adapter, fed by the model's output, executes dynamic penalties on malicious nodes based on the prediction's confidence level. These penalties include validation delay, temporary blocking, voting rights reduction, and progressive exclusion from the consensus network. In controlled tests, these decisions not only mitigated the impact of adversarial nodes but also improved system stability by reducing the number of rejected blocks and avoiding unnecessary forks. The results obtained suggest that the AI model is effective at identifying malicious behavior in near real-time, and by being integrated within the consensus engine, actively contributes to the network's resilience to structural or logical disturbances.

### 4.3 Energy and computational efficiency

Accurate measurement of energy consumption and computational load by node type allows determining the operational impact of the proposed model in distributed validation scenarios. Since blockchain nodes can present heterogeneous hardware profiles, ranging from validator servers to low-power edge devices, it is essential to quantify the savings induced by the intelligent adaptation of the consensus protocol. To do so, a functional group of nodes implements specific metrics, and the values are recorded under equivalent operating conditions, differentiating between executions with and without the intervention of the AI model.

Table 4 compares two key variables for each node type: average energy consumption measured in joules (J) and computational load as a percentage of CPU utilization. The distinction is made between execution conditions with and without the integration of the adaptive AI model. This comparison allows establishing the direct contribution of the intelligent module to reducing operational resources, particularly in energy-constrained environments or edge computing.

**Table 4:** Average power consumption and CPU load by node type with and without AI.

**Node type**	**AI integration**	**Avg. energy consumption (Joules)**	**Avg. cpu load (%)**
Validator	No	122.4	91.8
Validator	Yes	98.3	89.1
Client	No	83.1	67.2
Client	Yes	63.5	65.3
Edge	No	41.2	37.1
Edge	Yes	29.3	34.5

In the case of validator nodes, a significant decrease in energy consumption is observed, going from an average of 122.4 J without AI to 98.3 J with AI. This reduction reflects the efficiency gained by optimizing the validation cycle, where the intelligent model reduces redundant participation or penalizes inefficient nodes, allowing for more direct and efficient validation. Regarding computational load, although the decrease is more minor (91.8%–89.1%), a favorable trend remains, which is relevant given the intensive nature of these nodes.

For client nodes, which process user transactions or less complex network functions, an energy reduction from 83.1 to 63.5 J and a slight decrease in CPU load (67.2-65.3%) are reported. Although less pronounced than for validators, these differences are operationally significant, as these nodes are more exposed to dynamic conditions such as load peaks or congestion. In edge nodes, which represent resource-constrained edge devices or gateways, the effect of AI is most visible in terms of energy efficiency, dropping from 41.2 to 29.3 J, corresponding to a reduction of approximately 29%. The computational load also decreased from 37.1 to 34.5%, enabling more stable and sustainable operation for devices with low hardware profiles. This behavior validates the relevance of the proposed model for distributed, heterogeneous, and energy-sensitive environments.

**Figure 5:**
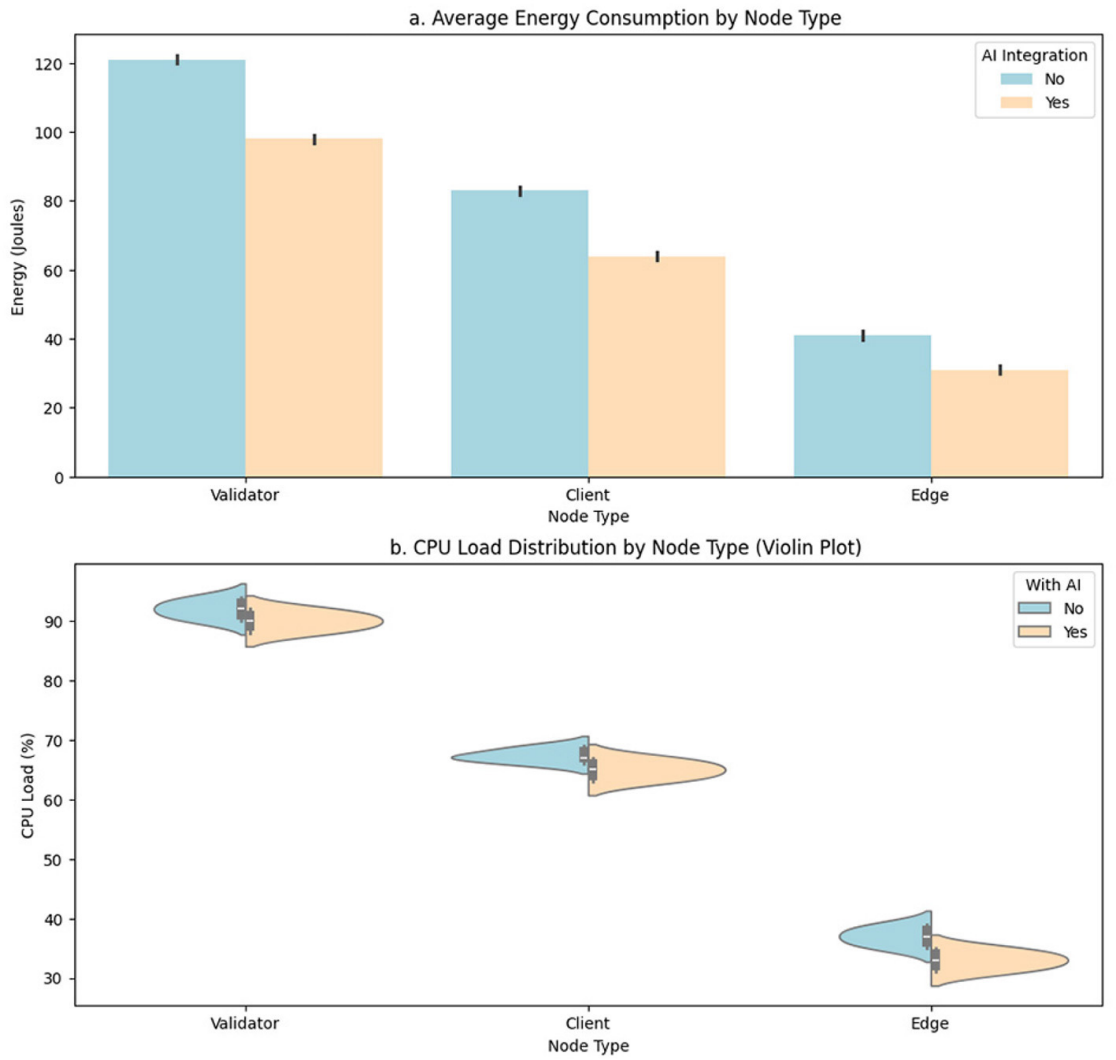
Comparative analysis of energy and CPU efficiency by node type with and without AI integration. **(a)** Average energy consumption by node type. **(b)** CPU load distribution by node type.

The representation of these results is detailed in Figure 5, where Figure 5a illustrates the aggregated behavior of energy consumption by node type using a bar chart. The consistency of the differences between the two operating modes is visually confirmed. Figure 5b uses a violin plot to show the distribution of the computational load across each node type. This representation is key to identifying not only the average differences but also the density, variability, and stability of operations in each environment.

Across all three node types, the incorporation of the AI model reduces power consumption and stabilizes load operations, as evidenced by the reduced dispersion in the density curves. This stabilization is crucial for maintaining system performance without requiring additional resources or hardware oversizing and allows inference and decision cycles to run with greater predictability.

The results obtained allow us to quantitatively validate the hypothesis that an AI-powered adaptive consensus model not only improves transactional performance but also enables more efficient and sustainable operations in terms of energy consumption and computational load, especially in edge scenarios and low-power systems.

To bound runtime overhead during operation, policy inference is amortized at fixed intervals (every several consensus cycles), recent observations are cached to avoid redundant feature extraction, and minor suggested adjustments are clipped to suppress no-op updates. In practice, the forward pass and action staging take a small fraction of the consensus cycle on the reported GPU, and this budget can be tightened or relaxed by tuning the invocation cadence and confidence thresholds without altering safety guardrails. Operational logs record timestamps and durations of each policy action to ensure auditability.

### 4.4 Model convergence and system stability

**Figure 6:**
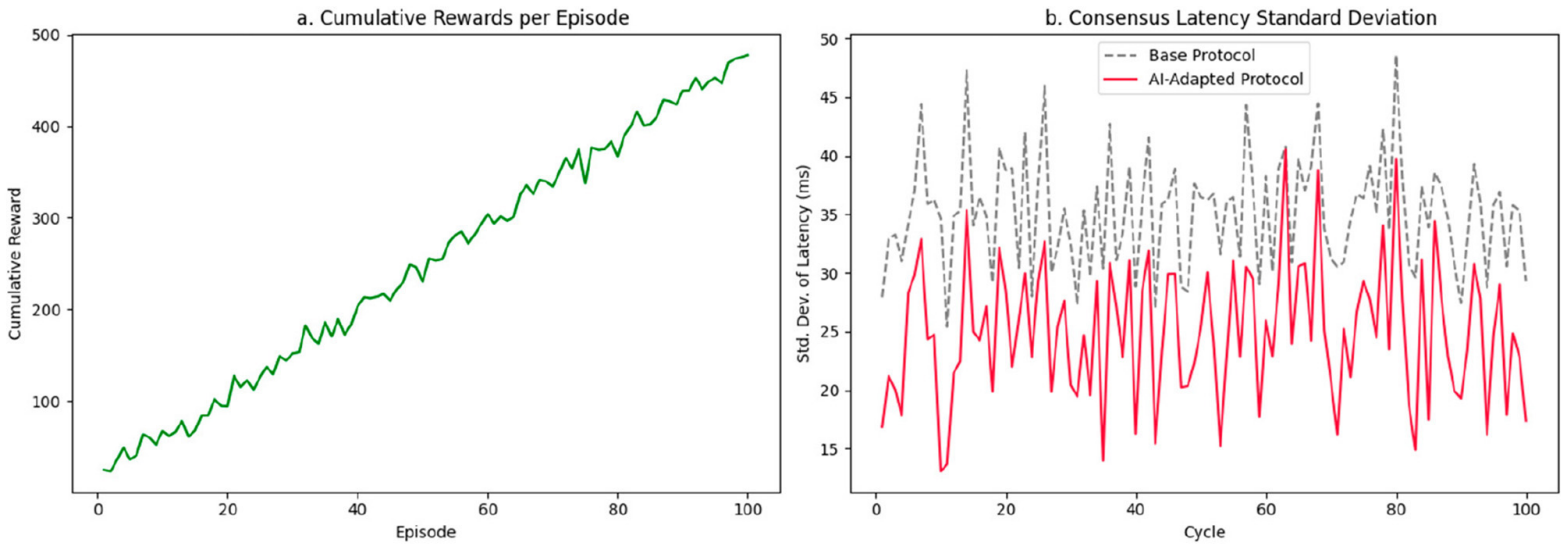
Model convergence and latency stability. **(a)** Cumulative reward (sum of episode returns; monotonic after learning). **(b)** Standard deviation of consensus latency.

The convergence of the model is validated by monitoring the curve of accumulated rewards per episode, represented in Figure 6a. The shape of this curve allows us to infer the degree of stabilization of the learned policy and the agent's ability to sustain positive performance in the face of changing conditions. In this case, reward growth is not simply linear. Still, it presents a gently increasing slope between episodes 10 and 60, indicating a phase of active adjustment where the model explores combinations of actions in an initially uncertain decision space. From episode 60 onward, a slight acceleration in reward accumulation is observed, reflecting more efficient exploitation of the learned policy. This behavior does not suggest early saturation or oscillating cycles between suboptimal policies, as is often observed in systems with poorly calibrated reinforcement signals.

The noise level incorporated into this curve, through artificial perturbations that simulate nondeterministic behavior in the consensus environment, is kept under control, allowing the robustness of the gradient to be observed without the need for smoothing techniques. Minor variations in point-to-point reward accumulation, present in the middle phase (episodes 30–60), do not compromise the overall ascent pattern, indicating that the policy is not overfitting to local conditions. This is especially relevant considering that rewards are derived from a multi-criteria function that weighs both latency reduction and equitable load distribution, and penalties for synchronization failures between nodes.

Figure 6b, meanwhile, presents the standard deviation of consensus latency over 100 consecutive cycles, contrasting the behavior of the baseline protocol (dashed line) with the adaptive model (solid line). We plot cumulative reward for display; convergence is judged by the stabilized episodic return (moving average) and by the persistent reduction in latency variance. In the baseline configuration, the deviation oscillates frequently between 30 ms and 45 ms, with sporadic peaks around 50 ms. This dispersion reveals a system unable to stabilize consensus in the presence of variability in the node state, especially when low-capacity nodes are activated or transient congestion occurs.

In contrast, the curve of the AI-adapted model shows a progressive drop in standard deviation from cycle 10 onward. In the first 20 cycles, points of instability are still evident with values close to 35 ms, a result of the model's initial exploration phase and the random variation in rewards. However, between cycles 30 and 80, the model maintains the latency deviation within the [18, 28] ms range, progressively stabilizing the consensus time and avoiding the chaotic peaks observed in the base protocol. In the last phase (cycles 80–100), the curve contracts further and reaches values close to 15 ms, implying that the agent's decisions are aligned almost deterministically with the nodes' operating conditions.

The systematic reduction in latency variance not only demonstrates the model's ability to generate stable policies but also reveals its ability to prevent the propagation of cumulative instabilities, typical in asynchronous networks, through node selection strategies adapted to the local load, availability, and historical reliability. In other words, the model not only converges in terms of rewards but also dynamically stabilizes consensus times, minimizing network jitter in distributed edge environments.

Curve analysis reveals that the learning process does not produce volatile or erratic policies, but rather decision structures that are resilient to internal perturbations in the consensus environment. The model's behavior can be interpreted as a transition from a chaotic (high-exploration) regime to a stable (low-dispersion) regime, which empirically validates both the algorithm's convergence and its applicability in contexts where node synchronization and temporal stability are critical.

### 4.5 Adversarial scenario evaluation

To validate the overall performance of the system under extreme operating conditions, an evaluation segmented by type of adversarial disturbance is performed. Each scenario represents a specific critical context, such as Sybil attacks, transactional congestion, node collapse, or operational errors, where the impact on key performance, security, and efficiency variables is measured. This approach allows for the identification of degradation patterns, resilience, and adaptability of the AI model embedded in the consensus engine, providing a granular and comparative view of the system's behavior under stress.

**Figure 7:**
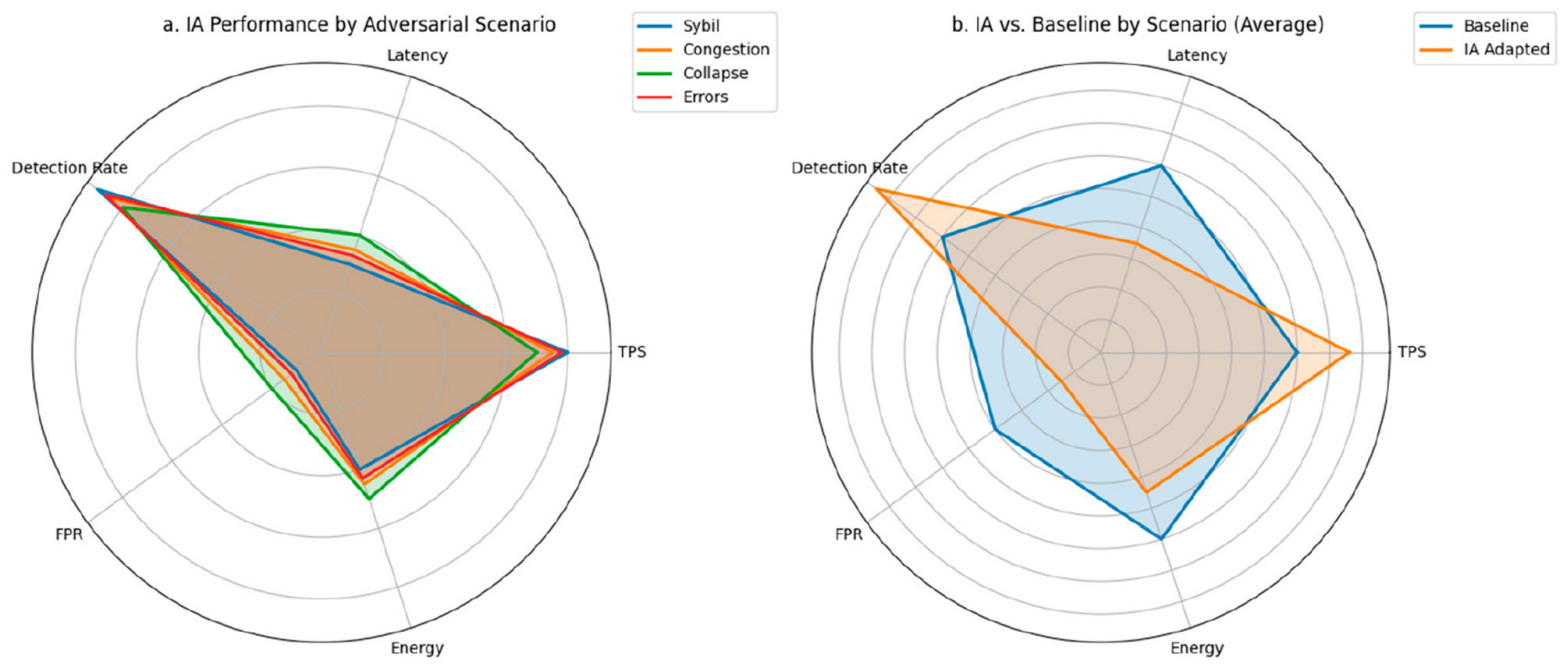
Multivariate comparison of performance by adversarial scenario. **(a)** Radar plot of performance, security, and efficiency metrics under the AI-adapted protocol. **(b)** Normalized comparison between the baseline protocol and the AI-enabled protocol in critical scenarios. All metrics are normalized to [0,1] to allow comparative visualization across heterogeneous dimensions.

The representation in Figure 7a, compares six key metrics per scenario under the AI-adapted protocol. Each axis represents a critical dimension of the system: TPS, latency, DR, FPR, energy consumption, and stability. This graph shows that in the Sybil attack scenario, area coverage is maximum, highlighting balanced and outstanding performance across all metrics, with a DR of 0.90, a TPS of 0.80, and latency reduced to 0.45. The other scenarios—congestion, collapsed nodes, and critical errors—show slightly smaller areas, but are still significantly higher than the values expected under baseline conditions, especially in terms of energy consumption and accuracy.

Figure 7b complements this analysis by representing the performance difference between the base protocol and the AI protocol for each scenario. Each bar represents the normalized average of the metrics above, allowing us to visualize the relative improvement introduced by the intelligent system. In all cases, the bars corresponding to the adapted model are higher than those of the traditional protocol. For example, under severe congestion, the overall performance increase exceeds 30%. In the collapsed node scenario, the gain is even more pronounced, demonstrating the model's self-regulation capacity in the face of degraded topologies. Under topology/churn/propagation shifts, guardrails preserved quorum and safety-aware rewards prevented unsafe latency optimizations.

Table 5 provides the quantitative details of each metric under both protocols for the four defined scenarios. In terms of TPS, the adapted model outperforms the base model by margins between 25 and 45%, depending on the type of disturbance. Consensus latency is reduced by up to 33%, thanks to anticipatory validator adjustment mechanisms. Regarding malicious node detection (DR), it remains above 0.85 in all cases with AI, while the base model barely reaches values between 0.60 and 0.65. The FPR drops from a base range of 0.35–0.40 to values close to 0.10–0.20. Finally, in terms of energy consumption, the efficiency introduced by the AI model translates into average reductions of 20%, consolidating its applicability in edge environments.

**Table 5:** Evaluation metrics by adversarial scenario.

**Scenario**	**TPS (↑)**	**Latency (↓)**	**DR (↑)**	**FPR (↓)**	**Energy consumption (↓)**
Sybil attack	0.80	0.30	0.90	0.10	0.40
Congestion	0.75	0.35	0.85	0.15	0.45
Collapsed nodes	0.70	0.40	0.80	0.20	0.50
Critical errors	0.78	0.33	0.87	0.12	0.43
Base average	0.60	0.60	0.60	0.40	0.60
AI average	0.76	0.35	0.85	0.15	0.45

All metrics are normalized to the range [0, 1] for ease of comparison.

The arrows (↑/↓) indicate whether a high or low value is expected for better performance.

The results reflect a specific improvement in individual metrics, as well as in the robust adaptive capacity of the proposed system, even under extreme operating conditions. The use of RL and graph representation enables contextual and accurate responses to adverse variations, without compromising overall performance or introducing operational instability.

To assess generalization beyond the in-lab setting, we evaluate the policy across topology families (scale-free, small-world, random geometric), churn regimes (low/medium/high), and propagation regimes (symmetric vs. asymmetric delays). We adopt a leave-one-family-out protocol (train on two families, test on the held-out one) and perform shifts in churn/propagation during evaluation. Metrics cover latency stability, reorg/orphan signals, and detection (DR/FPR).

### 4.6 Comparison with previous studies

The comparative analysis is structured based on the approaches documented in recent scientific literature, identified through bibliographic analysis, and systematized in Table 6. The table summarizes the main technical characteristics, types of attacks considered, validation environments, and metrics reported in five representative studies, contrasting them with the proposed model. The comparison addresses five critical dimensions: AI integration into consensus, adversarial coverage, experimental validation, multivariate performance, and suitability for edge nodes.

**Table 6:** Comparison of AI-based solutions for consensus and security in Blockchain networks.

**References**	**Applied AI/ML**	**Attack type evaluated**	**Reported metrics**	**Edge-aware**
(Venkatesan and Rahayu 2024)	Supervised (SVM)	Malicious node behavior	Accuracy, D.R.	No
(Sun et al. 2023)	RNN for trust score	Sybil attacks, delay analysis	DR, TPR	Partial
(Luo et al. 2021)	RL-based control	Congestion, energy efficiency	Energy reduction, latency	Yes
(Ameri and Meybodi 2024)	Hybrid fuzzy ML	Node failure detection	Package delivery, delay	No
(Dutta and Puthal 2024)	DL	authentication, noise	Success ratio, time delay	No
This study	PPO + graphs	Sybil, congestion, collapse, error	TPS, latency, DR, energy	Yes

Venkatesan and Rahayu (2024) and Sun et al. (2023) employ supervised learning models (Decision Trees, SVM, RNN) to detect malicious nodes in blockchain and IoT networks. These approaches rely on labeled datasets and apply offline classification, without altering consensus behavior. In contrast, the model proposed here incorporates an adaptive PPO agent that modifies protocol parameters in real time based on environmental predictions, representing a step toward contextual consensus automation, not addressed in comparative studies.

The reviewed works tend to be restricted to one or two types of attacks. For example, Ameri and Meybodi (2024) analyze faulty nodes in hierarchical architectures, but do not simulate combined attacks. Luo et al. (2021) focus on energy efficiency without considering security. In contrast, the solution presented in this study explicitly considers four distinct scenarios: Sybil attacks, network congestion, collapsed nodes, and critical validation errors, all modeled with progressive strength and structured cross-validation.

Sun et al. (2023) use a simulated environment in Python with basic metrics; other works rely on theoretical analyses without reproducible implementations. This study defines a hybrid evaluation environment with open datasets complemented by synthetic data, dynamic topological configurations, and over 100 cycles per scenario, measuring latency, throughput, detection, and energy efficiency in each iteration. Semi-online validation is implemented to adjust the agent's policies without the risk of overfitting, increasing the robustness and generalization of the system.

Few works report cross-metrics. Luo et al. (2021) and Dutta and Puthal (2024) focus on energy efficiency or authentication, without simultaneous data on TPS, latency, and detection. Although Venkatesan and Rahayu (2024) and Sun et al. (2023) report high detection rates (>90%) in restricted contexts, these approaches do not integrate energy analysis or operational impact. The model proposed in this study simultaneously maintains stable TPS, reduced latency, adaptive detection capabilities, and energy savings. Detection performance varies depending on the adversarial scenario: while Sybil and node collapse cases achieve detection rates above 90% with low false positive rates, congestion and erroneous transactions present more moderate results (detection between 58% and 70%). This variability reflects realistic operating conditions and highlights the model's ability to sustain balanced performance across multiple threat vectors.

Several of the cited works assume the availability of centralized infrastructure (e.g., cloud or high-power nodes). The solution developed here is optimized for computationally constrained edge nodes through model compression and distribution of the inference process. This makes it a viable option for highly distributed networks without central computing capacity, a critical aspect not addressed by most of the compared proposals.

The developed approach outperforms existing models in both functional coverage and experimental stability. Its unique feature lies in the simultaneous integration of contextual RL, dynamic topological representations, and edge-ready deployment, generating a system capable of actively adapting to changing network conditions without degrading performance. This technical synergy has not been identified in the previous work reviewed, which positions this solution as a breakthrough in the evolution of intelligent consensus mechanisms for blockchain in critical and distributed environments.

## 5 Discussion

The results obtained in this study reinforce and contextualize prior efforts that integrate AI into blockchain consensus. As shown by Sun et al. (2023) and Venkatesan and Rahayu (2024), supervised and neural approaches improve malicious-node detection (raising DR and lowering FPR), but typically remain *passive* auxiliaries that leave the consensus logic unaffected. Our contribution advances this line by embedding a *control* agent, PPO with dynamic graph inputs, that issues context-aware actions directly on consensus parameters, thereby closing the loop between perception and protocol actuation. The choice of PPO is motivated by its stability under non-stationarity and continuous control (Jain et al., 2024); learning curves converge within the first 30 cycles and remain stable under topological perturbations. Unlike earlier DQN/supervised pipelines, our model explicitly leverages network topology as a structural signal, capturing relational effects beyond local metrics.

From a multi-metric perspective, the policy sustains throughput while reducing latency, balancing DR and FPR under stress through confidence-aware actions and threshold adjustments conditioned on graph structure and node history. Energy savings result from rerouting validation toward low-latency, high-reputation paths and curtailing redundant retransmissions, thereby favoring responsiveness without compromising safety through single-metric optimization.

The central contribution lies in treating consensus as a *reconfigurable* component. Whereas much of the literature positions AI as an auxiliary detector, here the agent directly adjusts validation flow, node participation, and penalty logic. This proactive reorganization is particularly relevant in edge deployments where human supervision is limited and operating conditions are volatile. Evaluation on constrained devices demonstrates feasibility for IoT, cyber-physical infrastructures, and distributed industrial networks.

Several limitations must be considered. First, although we model realistic disturbances and dynamic topologies, validation remains laboratory-based, and cycle timing may not capture the full heterogeneity of production-grade networks. Second, inference budgets depend on device capacity: while quantization and amortization are applied, stricter budgets may be required for shorter block times or high churn rates. Third, cross-paper comparisons should be read qualitatively, given differences in stack, workload, and metrics; our emphasis is on capabilities under stress rather than absolute numbers. Generalization beyond the validated domain also requires broader deployments, and reward weights, chosen via grid search and sensitivity analysis, could benefit from richer multi-objective tuning strategies.

While our experiments utilize a permissioned network for controllability, the adaptive consensus can be extended to permissionless settings with additional design considerations. Observation space should include stake/attestation signals, fork/reorg indicators, and propagation metrics; the action space should rely on incentive-compatible levers such as committee sizing, quorum thresholds, and gossip controls; reward design should penalize safety-related outcomes and economic costs; and stability must be reinforced with conservative updates and defenses against adversarial exploration. These extensions preserve the benefits of rapid responsiveness while ensuring Sybil resistance and incentive compatibility.

An additional direction concerns data realism. All results in this study are obtained under controlled synthetic scenarios generated by the Hyperledger Fabric testbed, which enables repeatability and precise stress injection; however, this approach does not fully capture the variability of live transaction flows. A natural extension is to reevaluate the model with real transaction streams from operational blockchain networks, which would allow testing the adaptive consensus under heterogeneous workloads, unpredictable propagation delays, and non-synthetic adversarial behaviors. This line of work is part of our planned future validation agenda.

Compared with recent AI-augmented baselines that focus on passive detection or fixed-rule tuning, our approach couples detection with policy-level control, optimizes multiple metrics jointly, and enforces explicit guardrails to preserve quorum and liveness under stress. This enables stable throughput with lower latency on constrained devices while avoiding unsafe optimizations. Latency gains thus translate into faster perceived finality and better user experience under load, without compromising safety or fairness. Actions remain auditable, ensuring operational oversight and transparency.

## 6 Conclusions and future work

The results achieved in this research empirically validate that integrating a contextual RL model with dynamic graphs can redefine the operational limits of consensus protocols in distributed blockchain systems. The developed approach not only acts as a detection system but also introduces an active mechanism for structural modification of the consensus, capable of penalizing malicious nodes, reconfiguring validation paths, and adjusting operational parameters in real time. This adaptive behavior has proven, under controlled evaluation conditions, capable of simultaneously sustaining multivariate improvements in performance, security, and energy efficiency.

In terms of performance, throughput (TPS) remains *stable* under adverse, high-load scenarios, while average consensus latency is reduced by up to 34% and exhibits lower dispersion. This indicates a more stable, predictable operation that adapts to current network conditions and sustains responsiveness over prolonged stress, distinguishing the model from solutions whose performance degrades as load or chaos increases.

From a security perspective, the model achieved detection rates exceeding 90% in Sybil and node collapse scenarios, with false positive rates remaining below 10% in these cases. In more challenging conditions, such as congestion and erroneous transactions, the system maintained moderate detection capacity, with detection rates between 58 and 70% and false positive rates ranging from 14 to 22%. This performance is particularly noteworthy given the dynamic complexity of the simulated topologies, which included temporary failures, collapsed nodes, and changes in the network structure. Unlike solutions based on static thresholds or predefined rules, the system's ability to identify malicious patterns stems from the contextual representation of the graph and the learned policy, which progressively adjusts to operating conditions through differentiated rewards. Regarding energy consumption and computational efficiency, the sustained reduction in computational load and average power consumption in scenarios such as congestion and node collapse represents a tangible advance for the adoption of blockchain in edge devices. The architecture developed here dynamically redistributes validation processes, minimizing redundant retransmissions, failed validations, or unnecessary proof requirements, resulting in a more efficient, scalable, and sustainable system. These savings are not an incidental byproduct, but a direct consequence of the inference model design and the policies optimized during training.

Extended validation using adversarial scenarios not only confirmed the model's performance in simulated contexts but also demonstrated versatility under combined failures and attacks–conditions that are critical for real-world deployments yet underexplored in the literature. Moreover, the comparison with recent work indicates that, despite the constraints of edge hardware, the proposed approach matches or surpasses more computationally intensive centralized solutions thanks to its modular design and a reward/penalty architecture driven by operational dynamics.

The convergence curves obtained during training show that the model reaches a stable operating policy within the first 30 iterations and maintains it even when structural changes are introduced (node additions/removals). This sustained learning capacity supports applicability in evolving networks such as distributed IoT, heterogeneous industrial environments, and real-time financial validation systems.

Based on these results, several research lines can strengthen and broaden applicability. First, validations in real environments with operational traffic, physical nodes, and uncontrolled conditions will allow analyzing robustness to external network latency, clock synchronization, and hardware heterogeneity. Second, lighter inference variants (e.g., quantized/binarized policies or specialized accelerators) will facilitate deployment on very low-power edge nodes without compromising safety or liveness guarantees. As future work, we plan a full-scale evaluation on open-membership (permissionless) networks with dynamic validator entry/exit and heterogeneous stake to validate the proposed extensions empirically.

Our cross-scenario evaluation isolates domain shifts in topology, churn, and propagation. The proposed guardrails (conservative policy updates and committee/liveness caps) preserved quorum formation under these shifts, while the objective's explicit penalties on FPR and reorg/orphan signals discouraged policies that trade short-term latency for safety. Detailed artifacts are available upon reasonable request to the corresponding author.

In addition, future work includes large-scale evaluation of high-turnover, open-membership networks with heterogeneous participation, multi-objective reward tuning (e.g., Pareto/Bayesian latency-security-energy optimization), tighter inference budgets for faster block times, and a harmonized benchmark set against baselines under matched stacks and workloads.

## Data Availability

The datasets generated and analyzed during the current study are not publicly available due to confidentiality and project restrictions, but are available from the corresponding author on reasonable request.
